# Ethnic disparities in hospitalisation for COVID-19 in England: The role of socioeconomic factors, mental health, and inflammatory and pro-inflammatory factors in a community-based cohort study

**DOI:** 10.1016/j.bbi.2020.05.074

**Published:** 2020-08

**Authors:** Camille Lassale, Bamba Gaye, Mark Hamer, Catharine R. Gale, G David Batty

**Affiliations:** a Hospital del Mar Medical Research Institute (IMIM), Barcelona, Spain; b CIBER of Pathophysiology of Obesity and Nutrition (CIBEROBN), Instituto de Salud Carlos III, Madrid, Spain; c Department of Epidemiology and Public Health, University College London, UK; d Integrative Epidemiology of Cardiovascular Diseases, Paris Cardiovascular Research Center-INSERM U970 (PARCC), France; e Division of Surgery and Interventional Sciences, Faculty Medical Sciences, University College London, London, UK; f MRC Lifecourse Epidemiology Unit, University of Southampton, UK; g Lothian Birth Cohorts, Department of Psychology, University of Edinburgh, UK

**Keywords:** COVID-19, Ethnicity, Inflammatory factors

## Abstract

**Background:**

Differentials in COVID-19 hospitalisations and mortality according to ethnicity have been reported but their origin is uncertain. We examined the role of socioeconomic, mental health, and pro-inflammatory factors in a community-based sample.

**Methods:**

We used data on 340,966 men and women (mean age 56.2 years) from the UK Biobank study, a prospective cohort study with linkage to hospitalisation for COVID-19. Logistic regression models were used to estimate associations between ethnicity and hospitalisation for COVID-19.

**Results:**

There were 640 COVID-19 cases (571/324,306 White, 31/4,485 Black, 21/5,732 Asian, 17/5,803 Other). Compared to the White study members and after adjusting for age and sex, Black individuals had over a 4-fold increased risk of COVID-19 infection (odds ratio; 95% confidence interval: 4.32; 3.00–6.23), and there was a doubling of risk in the Asian group (2.12; 1.37, 3.28) and the ‘other’ non-white group (1.84; 1.13, 2.99). After controlling for potential explanatory factors which included neighbourhood deprivation, household crowding, smoking, body size, inflammation, glycated haemoglobin, and mental illness, these effect estimates were attenuated by 33% for Blacks, 52% for Asians and 43% for Other, but remained raised for Blacks (2.66; 1.82, 3.91), Asian (1.43; 0.91, 2.26) and other non-white groups (1.41; 0.87, 2.31).

**Conclusions:**

There were clear ethnic differences in risk of COVID-19 hospitalisation and these do not appear to be fully explained by measured factors. If replicated, our results have implications for health policy, including the targeting of prevention advice and vaccination coverage.

## Highlights

• Ethnic differentials in COVID-19 burden are reported but their origin is uncertain.
• In a UK cohort, Black and Asian individuals had a higher COVID-19 hospitalisation risk.
• Black individuals had more than a doubling of risk compared to White individuals.
• This held after adjusting for socioeconomic, physical and mental health confounders.
• The effect for the Asian group was diminished.
• Implications for health policy include targeting of prevention and vaccination.

## 1 Introduction

Ethnic disparities in health have traditionally been examined for non-communicable disease, chiefly obesity (Adult Obesity Facts, 2020, Public Health England, 2020), diabetes (Goff, 2019) and cardiovascular disease (George et al., 2017), however, there is emerging evidence that COVID-19 might disproportionately affect people from ethnic backgrounds. (Kirby, 2020, Aldridge et al., 2020) In the UK, inequalities in COVID-19 in prognostic studies have been reported such that, in cohorts of hospitalised patients, minority groups appear to have the greatest risk of progression to intensive care and death. (Williamson et al., 2020) In the US, a pooling of hospital data from 38 states also shows that minorities have a greater rate of deaths involving COVID-19 and this is particularly so for African-Americans. (Yancy, 2020, Research, 2020)

With neighbourhood deprivation and comorbidity only partially explaining these ethnic differentials, (Williamson et al., 2020) other causes need to be examined. These include individual socioeconomic status such as education, overrepresentation of minorities in in public-facing occupations, overcrowded living and working conditions, and greater prevalence of pro-inflammatory unhealthy lifestyle and chronic disease. (Kirby, 2020, Ross et al., 2020, Platt and Warwick, 2020, Centers for Disease Control and Prevention, 2019) Mental health problems, also more common in minorities, (NHS, 2014, Diaz-Venegas et al., 2016) may be related to infection and severity of respiratory infections via impaired innate and adaptive immunity. (Hamer et al., 2019, Gale et al., 2019) Finally, biological differences, such as impaired immunologic response functioning, (Webb Hooper and Napoles, 2020) are amplified in the present of racism and chronic stress.

With existing studies focusing on disease prognosis, it is unclear if people from ethnic groups also experience an elevated risk of disease onset, and, if so, what explains this burden. Accordingly, our aim was to assess the ethnic differences in serious cases of COVID-19 in a well-characterized, large, community-based cohort study in the UK, and investigate which underlying factors drive the observed associations.

## 2 Methods

### 2.1 Study population

We used data from UK Biobank, a prospective cohort study, the sampling and procedures of which have been well described. (Sudlow et al., 2015) Baseline data collection took place between 2006 and 2010 across twenty-two research assessment centres in the UK giving rise to a sample of 502,655 people aged 40 to 69 years (response rate 5.5%). (Sudlow et al., 2015) Ethical approval was received from the North-West Multi-centre Research Ethics Committee, and the research was carried out in accordance with the Declaration of Helsinki of the World Medical Association, and participants gave informed consent. For the present analysis, participants residing in Scotland and Wales were excluded as COVID-19 test data were only available for England.

### 2.2 Hospitalisation for COVID-19

Provided by Public Health England, data on COVID-19 status covered the period from 16th March to 26^th^April 2020 (http://biobank.ndph.ox.ac.uk/showcase/field.cgi?id = 40100), during which testing was largely restricted to those with symptoms in hospital. COVID-19 tests were performed on samples from combined nose/throat swabs using real time polymerase chain reaction (RT-PCR) in accredited laboratories. (NHS England and NHS, 2020) These data can therefore be regarded as a proxy for hospitalisations for severe COVID-19 cases.

### 2.3 Ethnicity

Ethnicity was self-reported at baseline assessment and based on 6 categories: White (including White British, White Irish, any other white background), Mixed (White and Black Caribbean, White and Black African, White and Asian, any other mixed background), Asian or Asian British (thereafter termed “Asian”, including Indian, Pakistani, Bangladeshi, any other Indian background), Black or Black British (“Black”, Caribbean, African, any other Black background), Chinese, and Other. To maintain statistical power in our analyses, we grouped together Chinese, Mixed and Other under the “Other” category.

### 2.4 Covariates

All variables were obtained at baseline and were grouped into 4 clusters.

### 2.5 Socioeconomic factors

Socioeconomic factors included highest educational attainment, household income, occupation, number of people living in the household, and the Townsend index of area deprivation (Townsend, 2017) (higher values denote deprivation). We created binary variables for education (university degree yes/no), total household income before tax (<18,000, ≥18,000 GBP), occupation (non-manual, manual). Size of the household had four groups (living alone; with two people; with three people; and four or more).

### 2.6 Lifestyle measures

Physical activity, smoking, and alcohol consumption were assessed by questionnaire. Participants were categorised into never, former, and current smokers. We grouped alcohol intake into three categories: never/rarely, and below or above current UK guidelines (≥14 units in women and ≥ 21 units in men). Leisure time physical activity was assessed using the short form version of the International Physical Activity Questionnaire (IPAQ). (Craig et al., 2003) Measuring duration and frequency of moderate-to-vigorous physical activity in the last week, data were grouped in 3 categories: inactive, somewhat active below the guidelines, and meeting activity guidelines (≥150 min/week moderate-to-vigorous physical activity or ≥ 75 min/week vigorous activity). (Nyberg et al., 2020)

### 2.7 Comorbidities

Body weight was measured using Tanita BC418MA scales and standing height using a Seca height measure, and body mass index (BMI) calculated [weight (kilograms)/height^2^ (meters^2^) squared].

Waist and hip circumference were measured with a non-elastic tape, and their ratio computed. The following self-reported physician diagnosed chronic diseases were used: cardiovascular diseases (heart attack, angina, stroke), chronic bronchitis and diabetes. Hypertension was defined as elevated measured blood pressure (≥140/90 mmHg) and /or use of anti-hypertensive medication. We used two indicators of mental health: contact with a psychiatrist for any disorder and symptoms of psychological distress as measured using the four-item version of the Patient Health Questionnaire (PHQ-4) in which scores ranged from 0 to 12 (categorised as 0, 1–2, ≥3 [high]). A verbal numerical reasoning task was used as a marker of cognitive function. (Gale et al., 2019)

### 2.8 Biomarkers

Non-fasting venous blood samples were drawn and assayed for C-reactive protein, glycated haemoglobin, and total and high-density lipoprotein cholesterol. (Mindell et al., 2012, Elliott and Peakman, 2008) Forced expiratory volume in 1 s, a marker of lung function, was quantified using spirometry with the best of three technically satisfactory exhalations used.

## 3 Statistical analyses

To compare participants’ characteristics between non-hospitalised and hospitalised patients, we performed t-tests for continuous variables and Chi-square tests for categorical variables. We fitted logistic regression models to estimate odds ratios and 95% confidence intervals for associations between ethnicity and hospitalisation for COVID-19. With the outcome being rare, odds ratios (OR) can be interpreted as relative risks. To quantify the contribution of factors to the ethnic differences, we used a simple approach to quantify the change in coefficient. Beginning with a comparator model where ORs were adjusted for age and sex, we subsequently fitted 5 models corresponding to groups of covariates: 1) socioeconomic, 2) lifestyle, 3) comorbidities, 4) biomarkers, and 5) all covariates. Percentage change in effect estimate was calculated as 100*(β_model x_ – β_base model_)/ β_base model_. With the aim being to compare attenuation of ORs by inclusion of various sets of factors, we selected all participants with non-missing values to run all five models. In a first sensitivity analysis, we present the estimates in samples with the maximum number of observations for each model. The cognitive function variable was only available in a subset of participants, therefore we present as a sensitivity analysis for the complete-case model with and without this factor. We also conducted the analysis separately for men and women. Finally, we also present results where covariates were imputed using multiple imputations by chain equations (Royston and White, 2011) with two datasets.

## 4 Results

Ethnicity data were available for 428,494 participants (235,528 women, 55%) who were alive prior to COVID-19 testing (up to 5 March 2020). The main analytical sample comprised 340,966 participants (640 COVID-19 cases) with complete data on the core set of covariates listed in Table 1, Table 2. As shown in Table 1, cases of COVID-19 were very slightly older and less likely to be female and highly educated. Hospitalised individuals more commonly lived in deprived neighbourhoods and had less favourable lifestyles as evidenced by the higher prevalence of physically inactive and cigarette smoking; cases were, however, less likely to drink alcohol. Patients also had a markedly higher prevalence of somatic comorbidities (hypertension, diabetes, cardiovascular disease, chronic bronchitis) and were somewhat more likely to report having seen a psychiatrist and have a higher level of psychological distress symptoms. Finally, cases displayed greater BMI, waist to hip ratio, CRP, and HbA1c levels, and lower HDL-cholesterol and lung function. White participants were underrepresented in hospitalised patients, whereas there were 3-times more Blacks and 2-times more Asians hospitalised with COVID-19.

**Table 1:** Baseline characteristics of participants according to COVID-19 hospitalisation, UK Biobank.

	Not hospitalised	Hospitalised	p-value
Number	427,594	900	
Ethnicity (%)			<0.001
Black	1.8	6.0	
Asian	2.2	5.1	
Other	1.9	3.1	
White	94.1	85.8	
Women (%)	55.0	44.4	<0.001
Age, years (mean, SD)	56.4 (8.1)	57.2 (9.0)	0.001
	*Percent*		
Higher education	32.6	26.0	0.001
Household ≥ 4 people	19.3	21.8	0.004
Neighborhood deprivation Highest quintile	19.6	33.0	<0.001
*Physical activity*			
Within guideline	53.9	49.4	
Active > 10 min not reaching guideline	27.9	24.2	
Inactive	18.2	26.3	<0.001
*Alcohol intake*			<0.001
Within guideline	36.0	28.5	
Never/rarely	31.4	41.7	
Heavy drinking	32.7	29.8	
*Cigarette Smoking*			<0.001
Never	55.4	46.7	
Past	34.6	41.9	
Current	10.0	11.4	
Hypertension	58.0	65.8	<0.001
Diabetes	5.0	9.9	<0.001
Cardiovascular disease	5.3	10.3	<0.001
Chronic bronchitis	1.4	3.1	<0.001
Seen a psychiatrist	11.4	15.7	<0.001
Psychological distress (PHQ4 ≥ 3)	23.7	28.6	0.001
	*Mean (SD)*		
BMI, kg/m^2^	27.4 (4.8)	29.1 (5.4)	<0.001
Waist to hip ratio	0.87 (0.09)	0.91 (0.09)	<0.001
C-reactive protein (mg/L)	2.51 (4.17)	3.50 (6.39)	<0.001
HbA1c (mmol/mol)	36.0 (6.6)	38.1 (8.9)	<0.001
Cholesterol (mmol/L)	5.70 (1.14)	5.43 (1.22)	<0.001
HDL-cholesterol (mmol/L)	1.45 (0.38)	1.32 (0.33)	<0.001
Forced expiratory volume in 1 sec (L)	2.82 (0.8)	2.70 (0.82)	<0.001

^a^p-value for Chi-squared test for categorical variables, and independent *t*-test for continuous variables

**Table 2:** Baseline characteristics of participants across ethnic groups, UK Biobank.

	Black	Asian	Other	White
Number	7734	9260	8304	403,196
COVID-19 cases (n, %)	31 (0.70)	21 (0.50)	17 (0.34)	571 (0.19)
Women (n, %)	4,507 (58.3)	4,350 (47.0)	5,015 (60.4)	221,656 (55.0)
Age, years (mean, SD)	51.8 (8)	53.2 (8.4)	52.1 (7.9)	56.6 (8.0)
	*Percent*			
Higher education	33.9	41.0	45.8	32.1
Household ≥ 4 people	31.9	51.7	32.2	18.1
Neighbourhood deprivation (Highest quintile)	63.7	37.8	44.2	17.9
Physical activity				
Meeting guideline	51.4	46.8	50.6	51.2
Active > 10 min not reaching guideline	28.2	29.3	28.6	27.9
Inactive	20.4	23.9	20.8	18.0
Alcohol intake				
Within guideline	24.9	19.5	24.3	36.8
Never/rarely	65.1	72.1	61.2	29.2
Heavy drinking	10.0	8.3	14.5	34.0
Smoking				
Never	70.7	77.6	60.4	54.5
Past smoker	17.3	13	25.7	35.6
Current smoker	12.0	9.4	13.8	9.9
Hypertension	62.4	56.4	49.3	58.2
Diabetes	11.1	16.8	7.9	4.5
Cardiovascular disease	4.6	7.8	4.0	5.3
Chronic bronchitis	0.6	0.9	0.9	1.4
Seen a psychiatrist	8.6	10.2	12.6	11.4
Psychological distress (PHQ4 ≥ 3)	36.5	41.7	36.2	22.9
	*Mean (SD)*			
BMI, kg/m^2^	29.5 (5.4)	27.2 (4.4)	27.0 (5.0)	27.4 (4.7)
Waist to hip ratio	0.87 (0.08)	0.9 (0.08)	0.87 (0.08)	0.87 (0.09)
C-reactive protein (mg/L)	2.78 (4.4)	2.79 (3.99)	2.37 (3.99)	2.50 (4.18)
HbA1c (mmol/mol)	39.3 (10.0)	40.5 (10.3)	37.5 (8.2)	35.8 (6.3)
Cholesterol (mmol/L)	5.25 (1.09)	5.33 (1.12)	5.53 (1.11)	5.72 (1.14)
HDL-cholesterol (mmol/L)	1.44 (0.36)	1.26 (0.32)	1.42 (0.38)	1.46 (0.38)
Forced expiratory volume in 1 sec (L)	2.33 (0.73)	2.23 (0.73)	2.54 (0.76)	2.85 (0.79)

A comment the resultsltsble of comparison between hospitalised and non-hospitalised participants Qll pvqlue

In Table 2 we show baseline characteristics according to ethnic groups. Despite being of younger age, compared to White participants, Black and Asian individuals experienced a higher prevalence of diabetes, higher levels of HbA1c and C-reactive protein and lower forced expiratory volume Blacks also had higher BMI and Asians higher waist to hip ratio. There was also an overrepresentation of people living in neighbourhoods characterised by greater deprivation and households of>4 people. By contrast, ethnic minority study members were more likely to avoid alcohol and cigarette smoking.

**Fig. 1:**
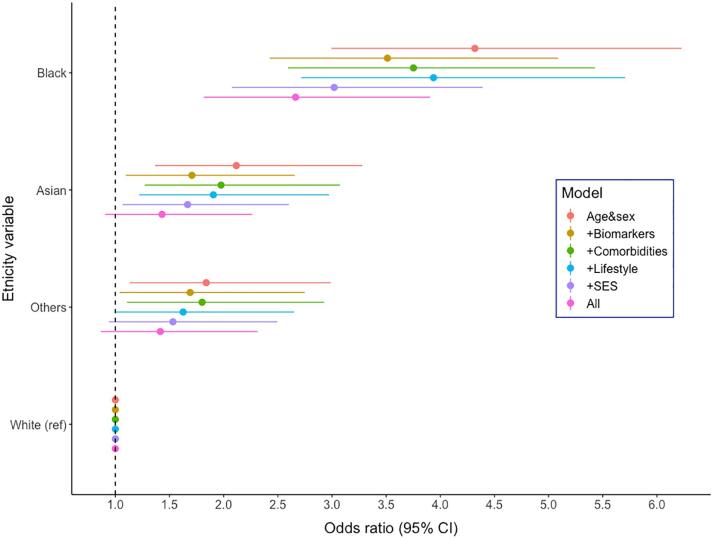
Association between ethnicity and hospitalisation for COVID-19 in UK Biobank (640 cases / 340,966 people at risk). Covariates included in each model. (1) Biomarkers: age, sex, log-CRP, log-HbA1c and total cholesterol. (2) Comorbidities: age, sex, cardiovascular disease, hypertension, diabetes, chronic bronchitis, body mass index and wait to hip ratio. (3) Lifestyle: age, sex, alcohol intake, physical activity, smoking. (4) Socioeconomic status: age, sex, Townsend deprivation index, education, number in household. (5) All: age, sex, Townsend deprivation index, education, number in household, alcohol intake, physical activity, smoking, cardiovascular disease, hypertension, chronic bronchitis, body mass index and wait to hip ratio, log-CRP, log-HbA1c, total cholesterol. Attenuation of coefficients was as follows: Black 1) −14.1%, 2) −9.6%, 3) −6.3%, 4) −24.4%, 5) –33.0%; Asian: 1) −28.7%, 2) −9.2%, 3) −14.1%, 4) –32.9%, 5) −52.2%; Others 1) −13.9%, 2) −3.4%, 3) −20.2%, 4) −30.0%, 5) −43.0%.

After adjusting for age and sex, compared to White participants, being from a Black ethnic background was associated with over a four-fold risk of hospitalisation for COVID-19 (odds ratio; 95% confidence interval: 4.32; 3.00–6.23), while a doubling was apparent in Asian (2.12; 1.37, 3.28) and Other ethnic groups (1.84; 1.13, 2.99) (Table 3). Gradual attenuation of the association after inclusion of groups of confounders can be seen in Fig. 1. The greatest attenuations were observed when socioeconomic factors were added to the multivariable model: 24.5% for Blacks, 31.9.3% for Asians, and 30.0% for Others. After further control for lifestyle factors, co-morbidities, and biomarkers of inflammatory disease (CRP, HbA1c and cholesterol), relationships were attenuated by 33.0% for Blacks, 52.2% for Asians and 43.0% for Others compared to the base model. There was, however, still evidence of associations, most obviously for Blacks (2.66; 1.82, 3.91). Effects for Asians (1.43; 0.91, 2.26) and Others (1.41; 0.87, 2.31), while raised, were not statistically significant at conventional levels (Table 2). In sex-specific analysis (Supplemental Table 1), we found that ORs for Black men (multivariable OR compared to white men: 3.51; 2.11, 5.81) were greater than for Black women (1.93; 1.07, 3.48, compared to white women). Contrarily, ORs for people from an Asian background were lower and weakened to a greater extent after inclusion of the full set of covariates for men than for women (attenuation by 72% for men, multivariable OR: 1.16; 0.60, 2.32, attenuation by 38% in women, OR 1.91; 1.01, 3.62).

**Table 3:** Multiply-adjusted odds ratios and 95% confidence intervals for the relation of baseline characteristics with hospitalisation for COVID-19 (640 cases / 340,966 people at risk).

	Age- and sex-adjusted			Multiply-adjusted a			
	OR	95% CI	p-value	OR	95% CI	p-value	Attenuation % c
Ethnicity (reference = White)							
Black (29 cases / 4,516)	4.32	(3.00–6.23)	<0.001	2.66	(1.82–3.91)	<0.001	–33.0
Asian (21 cases / 5,753)	2.12	(1.37–3.28)	0.001	1.43	(0.91–2.26)	0.125	−52.2
Other (17 cases / 5,820)	1.84	(1.13–2.99)	0.014	1.42	(0.87–2.31)	0.166	−43.0
Age (years)	1.02	(1.01–1.03)	0.001	1.02	(1.01–1.03)	0.003	
Male (ref = female)	1.56	(1.34–1.83)	<0.001	1.15	(0.92–1.44)	0.219	
Lower education (ref = university degree)				1.15	(0.96–1.37)	0.131	
Number in household (ref = 2 people)						0.001^b^	
One person				1.15	(0.93–1.43)	0.195	
3 people				1.22	(0.97–1.55)	0.093	
4 people or more				1.59	(1.26–2.01)	<0.001	
Townsend score (ref = least deprived, Q1)						<0.001^b^	
Q2				1.00	(0.76–1.33)	0.989	
Q3				0.99	(0.75–1.31)	0.937	
Q4				1.24	(0.95–1.62)	0.116	
Q5				1.67	(1.30–2.16)	<0.001	
Physical activity (ref = meeting guideline)						0.045^b^	
Active > 10 min not reaching guideline				0.93	(0.77–1.13)	0.466	
Inactive				1.22	(1.00–1.48)	0.049	
Alcohol (ref = within guideline)						0.041^b^	
Never/very rarely drink				1.30	(1.07–1.59)	0.01	
Intake above guideline				1.10	(0.90–1.34)	0.368	
Smoking (ref = never smoker)						0.008^b^	
Ex-smoker				1.30	(1.10–1.55)	0.003	
Current smoker				1.25	(0.96–1.62)	0.095	
Body mass index (kg/m^2^)				1.03	(1.02–1.05)	<0.001	
Waist-to-hip ratio (0.1 unit increase)				1.25	(1.09–1.42)	0.001	
Hypertension (ref = no)				0.98	(0.82–1.17)	0.84	
Cardiovascular disease (ref = no)				1.06	(0.79–1.42)	0.705	
Chronic bronchitis (ref = no)				1.34	(0.81–2.21)	0.259	
Ever seen a psychiatrist (ref = no)				1.24	(0.99–1.55)	0.057	
log-CRP (1 unit increase)				1.05	(0.92–1.19)	0.477	
log-HbA1c (1 unit increase)				1.60	(1.02–2.52)	0.043	
Cholesterol (mmol/L)				0.90	(0.84–0.97)	0.004	

a Estimates are all mutually adjusted,

b p-trend,

c Attenuation from the age and sex adjusted estimate to the multivariable adjusted estimate

In the maximum sample approach, the same pattern was observed (Supplemental Table 2). In a reduced sample of 116,990 individuals with available cognitive test score, associations were further attenuated after inclusion of this variable in the model, which displayed a strong association with COVID-19 hospitalisation (Supplemental Table 3). Finally, using multiple imputation, fully adjusted ORs were as follows: Black 2.53; 95% CI 1.87, 3.42; Asian 1.63; 1.17, 2.26; Others 1.44; 0.97, 2.12 (Supplemental Table 4).

## 5 Discussion

In a large community-dwelling cohort of over 400,000 individuals we found that ethnic minority groups in England experience a higher risk of COVID-19 hospitalisation. This effect was most pronounced in people of Black ethnic origin but risk was also raised for Asian individuals. The observed associations were attenuated but remained marked after adjustment for socioeconomic, lifestyle and health-related factors.

### 5.1 Mechanisms of effect

This work complements emerging prognostic data from various countries, in particular the USA and the UK, in large ethnically diverse populations, of disproportionately high rates of death involving COVID-19 in ethnic minority groups. (Aldridge et al., 2020, Centers for Disease Control and Prevention, 2019) There are several hypotheses that might explain these disparities. Firstly, minority ethnic groups are more likely to be in public-facing, service-based occupations which may mean they are less able to take effective physical distancing measures. Secondly, they are more likely to be of low income, in precarious contracts or self-employed, and to be living in intergenerational crowded households. (Aldridge et al., 2020) Moreover, if not legally resident, migrants may be fearful of accessing official health care services. (Ross et al., 2020) In the present analysis, we observed that household composition and neighbourhood deprivation are predictors of COVID-19 hospitalisation and partially attenuated the association between ethnicity and COVID-19.

It is also known that there are disparities in lifestyle and ill health - mental and physical - across ethnic groups, (Harris et al., 2006, Szczepura, 2005) which may explain susceptibility to a severe COVID-19 infection. However, although being important predictors, lifestyle, morbidity, biomarkers and mental health only partially diminished the association between the infection and ethnicity. Markers of central (waist to hip ratio) and general adiposity (BMI) were strongly related to COVID-19 hospitalisation, and unfavourable levels of these adiposity indices are more common in the Black population, (Public Health England, 2020) however, taking them into account did not eliminate ethnic differences in the infection. Adding biomarkers into the model also had some explanatory power, particularly in men, mostly due to the high prevalence of diabetes and elevated HbA1c in the Asian population, (Goff, 2019) and the presence of low grade inflammation as evidenced by higher C-reactive protein levels. Another potentially important result is the strength of the association between mental illness and COVID-19, and how taking into account cognitive function attenuated the association across all ethnic groups. However, markers of mental health, alongside inflammation, which may result from racism or other stressors experienced more often by ethnic minority, did not fully explain the association, although specific measures of chronic stress and discrimination would have had greater utility.

### 5.2 Study strengths and limitations

This is the first study of disease onset in the context of ethnic inequalities in COVID-19 and one which takes into account an extensive set of potential confounders and mediators, spanning individual and neighbourhood socioeconomic factors, lifestyle and markers of mental and physical health. The study has other strengths, including being based on a well-characterized large community-based sample. Additionally, study members were linked to objective measurement of the disease as opposed to self-report with confirmation of COVID-19 status being based on biological samples using PCR methodology, considered to be the gold standard. The study is not without its weaknesses. First, due to the absence of systematic testing across the UK, these data come from hospital records, therefore reflect only patients with a manifestation of the disease severe enough to require inpatient admission into hospital. Some cases of COVID-19 could also have been captured in patients originally hospitalised for reasons other than the infection. Second, the UK Biobank cohort is not representative of the general UK population. Therefore, absolute prevalence and risks should not be interpreted as such, but an aetiological investigation of risk factor association such as the present study are likely to be generalizable. (Batty et al., 2020) However, it is important to keep in mind that double selection of the sample – UK Biobank participants are not representative from the general population, and we selected a non-missing analytical sample within the cohort – may lead to collider bias. (Griffith et al., 2020) This means that conditioning on factors associated with the selection of the sample can distort or induce spurious associations. For example, this is likely to have been the case in studies finding that current smokers appear protected against COVID-19. (Simons et al., 2020) In the present study, smoking (in particular ex-smokers) was associated with greater risk of COVID-19 hospitalisation, somewhat ruling out collider bias. Third, despite using an extensive set of socioeconomic factors, both at individual and area level, we failed to capture some features that may be particularly relevant to the ethnic differences observed in the COVID-19 pandemic context: occupation did not classify between public facing occupations, not only health professionals, but also supermarket clerks, bus drivers or couriers. The number of people in the household, while a proxy for overcrowding, does not capture intergenerational co-living. Also, markers of mental health were not specific to racism or discrimination. Finally, exposure data were collected a few years ago (2006–2010) and participants’ health and living circumstances may have changed. Also, we excluded study members who had died prior to 5th March 2020 because they could not contribute to the risk set, however, ascertainment of COVID-19 hospitalisation did not reliably begin until 16th March. It is unlikely, however, that the absence of vital status data for this 11-day period would have biased our effect estimates.

## 6 Conclusions

In England, the observed ethnic disparities in hospitalisation for COVID-19 was strong, in particular comparing Black and White individuals, and to a lower extent for Asian individuals too, and not fully explained by an extensive set of factors spanning socioeconomic, lifestyle and inflammatory disease disparities. If replicated, this has implications for health policy, including the targeting of prevention advice and vaccination coverage. Further research is needed to better understand the underlying mechanisms driving the racial/ethnic disparities in hospitalisation for COVID-19 observed in our study.

## Funding

CL is supported by the Beatriu de Pinós postdoctoral programme of the Government of Catalonia's Secretariat for Universities and Research of the Ministry of Economy and Knowledge (2017-BP-00021). GDB is supported by the UK Medical Research Council (MR/P023444/1) and the US National Institute on Aging (1R56AG052519-01; 1R01AG052519-01A1); There was no direct financial or material support for the work reported in the manuscript.

## Declaration of Competing Interest

The authors declare that they have no known competing financial interests or personal relationships that could have appeared to influence the work reported in this paper.
