# Supplementary material for: Ethnic disparities in hospitalisation for COVID-19 in England: The role of socioeconomic factors, mental health, and inflammatory and pro-inflammatory factors in a community-based cohort study
Source: Brain Behav Immun. 2020 Aug;88:44–9. doi: 10.1016/j.bbi.2020.05.074 (PMC7263214; doi:10.1016/j.bbi.2020.05.074)
Supplement: Supplementary data 1 [file mmc1.docx]

Supplemental Table 1. Association between ethnicity and hospitalization for COVID-19 in UK Biobank – according to sex

|  |  |  | Men n=155,913 | |  |  |  |  | Women n=185,053 | |  |  |
| --- | --- | --- | --- | --- | --- | --- | --- | --- | --- | --- | --- | --- |
|  |  | Cases | OR | 95% CI | p-value | % Attenuation | Cases | OR | 95% CI | p-value | % Attenuation |  |
| Black | Age&sex | 18 | 5.41 | (3.34 - 8.76) | <0.001 |  | 13 | 3.33 | (1.9 - 5.85) | <0.001 |  |  |
| Black | +SES |  | 3.64 | (2.22 - 5.97) | <0.001 | -23.4 |  | 2.44 | (1.37 - 4.34) | 0.002 | -25.8 |  |
| Black | +Lifestyle |  | 4.94 | (3.03 - 8.06) | <0.001 | -5.3 |  | 2.97 | (1.68 - 5.25) | <0.001 | -9.6 |  |
| Black | +Comorbidities | | 5.41 | (3.33 - 8.79) | <0.001 | 0.0 |  | 2.49 | (1.41 - 4.40) | 0.002 | -24.3 |  |
| Black | +Biomarkers | | 4.48 | (2.75 - 7.30) | <0.001 | -11.2 |  | 2.73 | (1.54 - 4.84) | 0.001 | -16.5 |  |
| Black | All |  | 3.51 | (2.11 - 5.81) | <0.001 | -25.7 |  | 1.93 | (1.07 - 3.48) | 0.029 | -45.5 |  |
| Asian | Age&sex | 10 | 1.68 | (0.89 - 3.15) | 0.109 |  | 11 | 2.84 | (1.54 - 5.20) | 0.001 |  |  |
| Asian | +SES |  | 1.32 | (0.69 - 2.51) | 0.398 | -46.3 |  | 2.27 | (1.22 - 4.20) | 0.009 | -21.5 |  |
| Asian | +Lifestyle |  | 1.51 | (0.80 - 2.86) | 0.209 | -20.7 |  | 2.53 | (1.36 - 4.71) | 0.003 | -10.8 |  |
| Asian | +Comorbidities | | 1.65 | (0.87 - 3.12) | 0.124 | -3.3 |  | 2.49 | (1.35 - 4.59) | 0.004 | -12.6 |  |
| Asian | +Biomarkers | | 1.35 | (0.71 - 2.55) | 0.359 | -42.4 |  | 2.33 | (1.26 - 4.31) | 0.007 | -18.9 |  |
| Asian | All |  | 1.16 | (0.60 - 2.23) | 0.669 | -72.1 |  | 1.91 | (1.01 - 3.62) | 0.047 | -37.9 |  |
| Others | Age&sex | 10 | 2.42 | (1.28 - 4.55) | 0.006 |  | 7 | 1.33 | (0.62 - 2.82) | 0.462 |  |  |
| Others | +SES |  | 1.95 | (1.03 - 3.69) | 0.040 | -24.2 |  | 1.15 | (0.54 - 2.45) | 0.725 | -51.8 |  |
| Others | +Lifestyle |  | 2.11 | (1.12 – 4.00) | 0.021 | -15.1 |  | 1.17 | (0.55 - 2.49) | 0.687 | -44.8 |  |
| Others | +Comorbidities | | 2.45 | (1.30 - 4.61) | 0.006 | 1.4 |  | 1.25 | (0.59 - 2.66) | 0.562 | -21.1 |  |
| Others | +Biomarkers | | 2.19 | (1.16 - 4.13) | 0.016 | -11.2 |  | 1.25 | (0.59 - 2.67) | 0.557 | -20.0 |  |
| Others | All |  | 1.82 | (0.95 - 3.46) | 0.069 | -32.4 |  | 1.04 | (0.48 - 2.23) | 0.925 | -86.8 |  |
|  | | | | | | | | | | | | |

Supplemental Table 2. Association between ethnicity and hospitalization for COVID-19 in UK Biobank – maximum sample for each set of covariates

|  |  | N total sample | OR | 95% CI | Attenuation (%) |
| --- | --- | --- | --- | --- | --- |
| Black | Age&sex | 428,494 | 4.07 | (3.08 - 5.39) |  |
| Black | +SES | 417,511 | 2.65 | (1.94 - 3.61) | -30.6 |
| Black | +Lifestyle | 396,353 | 3.74 | (2.76 - 5.08) | -6.0 |
| Black | +Comorbidities | 415,705 | 3.95 | (2.97 - 5.26) | -2.2 |
| Black | +Biomarkers | 377,117 | 3.35 | (2.40 - 4.69) | -13.9 |
| Black | All | 341,593 | 2.63 | (1.79 - 3.86) | -31.2 |
| Asian | Age&sex | 428,494 | 2.68 | (1.99 - 3.62) |  |
| Asian | +SES | 417,511 | 2.10 | (1.52 - 2.90) | -24.8 |
| Asian | +Lifestyle | 396,353 | 2.25 | (1.58 - 3.19) | -18.0 |
| Asian | +Comorbidities | 415,705 | 2.47 | (1.79 - 3.42) | -8.4 |
| Asian | +Biomarkers | 377,117 | 1.87 | (1.31 - 2.67) | -36.8 |
| Asian | All | 341,593 | 1.42 | (0.90 - 2.25) | -64.5 |
| Others | Age&sex | 428,494 | 1.97 | (1.35 - 2.88) |  |
| Others | +SES | 417,511 | 1.49 | (0.99 - 2.26) | -40.7 |
| Others | +Lifestyle | 396,353 | 1.70 | (1.13 - 2.57) | -21.3 |
| Others | +Comorbidities | 415,705 | 1.74 | (1.15 - 2.65) | -17.8 |
| Others | +Biomarkers | 377,117 | 1.52 | (0.96 - 2.41) | -38.1 |
| Others | All | 341,593 | 1.40 | (0.86 - 2.29) | -50.1 |

Supplemental Table 3. Association between ethnicity and hospitalization for COVID-19 in UK Biobank – based on subgroup with data on verbal numerical reasoning

|  | Age- and sex-adjusted | | | Multiply-adjusted a | | | Multiply-adjusted + cognition | | |
| --- | --- | --- | --- | --- | --- | --- | --- | --- | --- |
| N cases / N total  206 / 116,990 | OR | 95% CI | p-value | OR | 95% CI | p-value |  |  |  |
| Ethnicity (reference=White) | | |  |  |  |  |  |  |  |
| Black (13/ 2,013) | 4.61 | (2.6 - 8.17) | <0.001 | 3.63 | (1.96 - 6.74) | <0.001 | 3.06 | (1.64 - 5.73) | <0.001 |
| Asian (7/ 2,738) | 1.64 | (0.77 - 3.49) | 0.204 | 1.15 | (0.51 - 2.59) | 0.730 | 0.94 | (0.42 - 2.14) | 0.889 |
| Other (7/ 2,311) | 2.13 | (1.00 - 4.56) | 0.051 | 1.74 | (0.8 - 3.79) | 0.163 | 1.47 | (0.67 - 3.24) | 0.335 |
| Age (years) | 1.02 | (1.01 - 1.04) | 0.013 | 1.02 | (0.99 - 1.04) | 0.182 | 1.02 | (0.99 - 1.04) | 0.204 |
| Male (vs female) | 1.66 | (1.25 - 2.19) | <0.001 | 0.93 | (0.6 - 1.45) | 0.758 | 0.94 | (0.6 - 1.46) | 0.768 |
| **Verbal numerical reasoning** | | |  |  |  |  | 0.90 | (0.83 - 0.96) | 0.003 |
| Lower education | |  |  | 1.06 | (0.78 - 1.44) | 0.695 | 0.93 | (0.68 - 1.28) | 0.659 |
| Household composition (ref=2 people) | | | |  |  |  |  |  |  |
| One person | |  |  | 1.17 | (0.8 - 1.7) | 0.413 | 1.16 | (0.8 - 1.69) | 0.429 |
| 3 people |  |  |  | 1.18 | (0.78 - 1.78) | 0.427 | 1.18 | (0.78 - 1.78) | 0.432 |
| 4 people or more | |  |  | 1.27 | (0.84 - 1.93) | 0.265 | 1.27 | (0.84 - 1.93) | 0.264 |
| Townsend deprivation (ref=least deprived Q1) | | | | |  |  |  |  |  |
| Q2 |  |  |  | 1.12 | (0.71 - 1.76) | 0.640 | 1.11 | (0.7 - 1.75) | 0.660 |
| Q3 |  |  |  | 1.03 | (0.65 - 1.64) | 0.896 | 1.01 | (0.64 - 1.61) | 0.957 |
| Q4 |  |  |  | 0.90 | (0.56 - 1.44) | 0.646 | 0.88 | (0.55 - 1.41) | 0.589 |
| Q5 |  |  |  | 1.06 | (0.67 - 1.68) | 0.793 | 1.01 | (0.64 - 1.6) | 0.965 |
| Physical activity (ref=meeting guideline) | | | |  |  |  |  |  |  |
| Active >10min not reaching guideline | | | | 0.96 | (0.69 - 1.35) | 0.826 | 1.00 | (0.71 - 1.4) | 0.987 |
| Inactive |  |  |  | 1.46 | (1.04 - 2.05) | 0.031 | 1.48 | (1.05 - 2.08) | 0.025 |
| Alcohol (ref=within guideline) | | |  |  |  |  |  |  |  |
| Never/very rarely drink | | |  | 1.62 | (1.13 - 2.31) | 0.009 | 1.58 | (1.11 - 2.26) | 0.012 |
| Dangerous intake above guideline | | | | 1.36 | (0.94 - 1.96) | 0.102 | 1.39 | (0.96 - 2) | 0.083 |
| Smoking (ref=never smoker) | | |  |  |  |  |  |  |  |
| Ex-smoker | |  |  | 1.35 | (0.99 - 1.84) | 0.057 | 1.36 | (1 - 1.85) | 0.053 |
| Current smoker | |  |  | 1.50 | (0.95 - 2.35) | 0.080 | 1.47 | (0.94 - 2.32) | 0.092 |
| Body mass index (kg/m^2^) | | |  | 1.00 | (0.97 - 1.03) | 0.957 | 1.00 | (0.97 - 1.04) | 0.994 |
| Waist-to-hip ratio (+0.1 unit) | | |  | 1.45 | (1.15 - 1.84) | 0.002 | 1.44 | (1.13 - 1.82) | 0.003 |
| Hypertension | |  |  | 1.13 | (0.82 - 1.56) | 0.453 | 1.12 | (0.81 - 1.55) | 0.483 |
| Cardiovascular disease | | |  | 0.98 | (0.58 - 1.66) | 0.939 | 0.97 | (0.57 - 1.64) | 0.898 |
| Chronic bronchitis | |  |  | 2.39 | (1.1 - 5.2) | 0.028 | 2.34 | (1.08 - 5.09) | 0.032 |
| Ever seen a psychiatrist | | |  | 1.32 | (0.89 - 1.95) | 0.164 | 1.30 | (0.88 - 1.92) | 0.186 |
| log-CRP |  |  |  | 1.08 | (0.87 - 1.36) | 0.483 | 1.08 | (0.86 - 1.36) | 0.494 |
| log-HbA1c | |  |  | 1.46 | (0.65 - 3.28) | 0.353 | 1.46 | (0.65 - 3.27) | 0.357 |
| Cholesterol (mmol/L) | | |  | 0.86 | (0.75 - 0.98) | 0.026 | 0.86 | (0.75 - 0.98) | 0.027 |
| HDL-cholesterol (mmol/L) | | |  | 0.65 | (0.39 - 1.09) | 0.103 | 0.64 | (0.38 - 1.08) | 0.094 |
| Forced expiratory volume (L) | | |  | 0.94 | (0.54 - 1.64) | 0.829 | 0.99 | (0.56 - 1.75) | 0.979 |

Supplemental Table 4. Association between ethnicity and hospitalization for COVID-19 in UK Biobank - using multiple imputation for covariates

|  | Age and sex | | | All predictors | | |  |
| --- | --- | --- | --- | --- | --- | --- | --- |
| N cases / N total  900 / 428,494 | OR | 95% CI | p-value | OR | 95% CI | p-value | Attenuation (%) |
| Ethnicity (reference=White) |  |  |  |  |  |  |  |
| Black (54 / 7,734) | 4.07 | (3.08 - 5.39) | <.001 | 2.61 | (1.93 - 3.55) | <.001 | -31.6 |
| Asian (46 / 9,260) | 2.68 | (1.99 - 3.62) | <.001 | 1.68 | (1.20 - 2.34) | 0.003 | -47.7 |
| Other (28 / 8,304) | 1.97 | (1.35 - 2.88) | <.001 | 1.40 | (0.93 - 2.09) | 0.105 | -50.7 |
| Age (years) | 1.02 | (1.01 - 1.03) | <.001 | 1.02 | (1.01 - 1.03) | 0.004 |  |
| Male (vs female) | 1.52 | (1.33 - 1.73) | <.001 | 1.15 | (0.93 - 1.42) | 0.194 |  |
| Lower education |  |  |  | 1.14 | (0.96 - 1.34) | 0.125 |  |
| Household composition (ref=2 people) |  |  |  |  |  |  |  |
| One person |  |  |  | 1.10 | (0.91 - 1.33) | 0.319 |  |
| 3 people |  |  |  | 1.13 | (0.92 - 1.39) | 0.236 |  |
| 4 people or more |  |  |  | 1.49 | (1.22 - 1.82) | <.001 |  |
| Townsend deprivation (ref=least deprived Q1) | | |  |  |  |  |  |
| Q2 |  |  |  | 1.12 | (0.87 - 1.43) | 0.383 |  |
| Q3 |  |  |  | 1.13 | (0.88 - 1.44) | 0.339 |  |
| Q4 |  |  |  | 1.33 | (1.05 - 1.68) | 0.017 |  |
| Q5 |  |  |  | 1.69 | (1.34 - 2.13) | <.001 |  |
| Low income (Q1 vs others) |  |  |  | 1.15 | (0.93 - 1.42) | 0.179 |  |
| Manual occupation (vs non-manual) |  |  |  | 1.05 | (0.88 - 1.26) | 0.555 |  |
| Physical activity (ref=meeting guideline) |  |  |  |  |  |  |  |
| Active >10min not reaching guideline |  |  |  | 0.95 | (0.79 - 1.14) | 0.547 |  |
| Inactive |  |  |  | 1.20 | (1.02 - 1.42) | 0.031 |  |
| Alcohol (ref=within guideline) |  |  |  |  |  |  |  |
| Never/very rarely drink |  |  |  | 1.27 | (1.07 - 1.51) | 0.007 |  |
| Intake above guideline |  |  |  | 1.21 | (1.01 - 1.44) | 0.037 |  |
| Smoking (ref=never smoker) |  |  |  |  |  |  |  |
| Ex-smoker |  |  |  | 1.31 | (1.13 - 1.52) | <.001 |  |
| Current smoker |  |  |  | 1.01 | (0.80 - 1.28) | 0.913 |  |
| Body mass index (kg/m^2^) |  |  |  | 1.02 | (1.00 - 1.03) | 0.026 |  |
| Waist-to-hip ratio (+0.1 unit) |  |  |  | 1.21 | (1.08 - 1.36) | 0.001 |  |
| Hypertension |  |  |  | 1.02 | (0.87 - 1.19) | 0.814 |  |
| Cardiovascular disease |  |  |  | 1.09 | (0.85 - 1.39) | 0.49 |  |
| Chronic bronchitis |  |  |  | 1.52 | (1.03 - 2.24) | 0.034 |  |
| Psychological distress (ref= low) |  |  |  |  |  |  |  |
| Moderate |  |  |  | 1.20 | (1.00 - 1.44) | 0.053 |  |
| High |  |  |  | 1.14 | (0.95 - 1.36) | 0.163 |  |
| Ever seen a psychiatrist |  |  |  | 1.25 | (1.04 - 1.51) | 0.019 |  |
| log-CRP |  |  |  | 1.10 | (0.99 - 1.23) | 0.075 |  |
| log-HbA1c |  |  |  | 1.30 | (0.86 - 1.95) | 0.213 |  |
| Cholesterol (mmol/L) |  |  |  | 0.69 | (0.53 - 0.89) | 0.004 |  |
| HDL-cholesterol (mmol/L) |  |  |  | 0.94 | (0.86 - 1.02) | 0.121 |  |
| Forced expiratory volume (L) |  |  |  | 0.82 | (0.65 - 1.04) | 0.109 |  |
